# Pathogen identification of *Gentiana macrophylla* root-knot nematode disease in Yulong, China

**DOI:** 10.21307/jofnem-2021-056

**Published:** 2021-06-12

**Authors:** Wentao Wu, Shanshan Xu, Zewen Gao, Shusheng Zhu, Youyong Zhu, Yang Wang, Xiahong He

**Affiliations:** 1 Key Laboratory of Agricultural Biodiversity and Pest Control, College of Plant Protection, Yunnan Agricultural University, Yunnan, Kunming, 650201, China; 2 School of Landscape and Horticulture, Southwest Forestry University, Yunnan, Kunming 650233, China

**Keywords:** *Gentiana macrophylla*, *Meloidogyne hapla*, Pathogen, Identification

## Abstract

In September 2020, samples of galled roots with rhizosphere soil were collected from declining *Gentiana macrophylla* in Yulong County, China. The pathogenic nematodes were identified by observing morphological characteristics of females, second-stage juveniles and perineal pattern, sequence alignments, and specific amplification of sequence characterized amplified region (SCAR). The results showed that the perineal pattern of this nematode was round or oval, the dorsal arch was moderately high or low, one side or both of the lateral field extended to form a wing shape, the tail region had punctations, and the morphological characteristics and morphometric values of second-stage juveniles and females were similar to those of *Meloidogyne hapla*. The ITS region fragment of this nematode were highly similar to those of *M. hapla* in NCBI database, with a similarity of over 99.35%. Using the SCAR specific primers, a specific band with an expected size of approximately 440 bp was amplified from this nematode. Morphological and molecular identification supports the nematode species found on Gentiana macrophylla as *M. hapla*. This is the first report of this regulated root-knot nematode on *Gentiana macrophylla* in China.

Qinjiao (*Gentiana macrophylla*) is a perennial herb of the Gentianaceae family, mainly produced in Mongolia, Russia, and China. *Gentiana macrophylla* has been widely cultivated in the southwest region of China for medicinal uses (Zhang et al., 2003). Severely stunted and withered Qinjiao plants with rotted and galled roots were observed in a field of the Yulong country (N 99°46′; E 27°18′) in September 2020. These are typical symptoms of infection by root-knot nematodes (RKN; *Meloidogyne spp.*). In order to clarify the nematode species of *Gentiana macrophylla*, extracted root-knot nematodes were identified by both molecular and morphological methods as *Meloidogyne hapla*.

## Materials and methods

The nematodes were collected from the soil in the root zone using standard procedures (Hooper, 1990). For morphological studies, the nematodes were killed with hot water, fixed in 5% formalin solution, and mounted in glycerin slides using the Seinhorst technique (Seinhorst, 1959). For molecular studies, DNA was extracted from females according to Blok et al. (1997). In species identification, two sets of primers were used for the amplification: species-specific SCAR primers JMV1/JMV hapla (Adam et al., 2007) and ITS region primers 18S/26S (Vrain et al., 1992). Amplifications were performed in a PCR Thermal Cycler (TaKaRa, China). The amplifications sequences were submitted to the GenBank database under accession numbers MW897745. In addition, to verify Koch postulates, *Gentiana macrophylla* seedlings (*n* = 10, 2-3 leaves stage) were infested with 1,500 *M. hapla* J2 and maintained at 20 to 25°C in a greenhouse. The test was repeated three times with an inoculation with sterile water as a control.

## Results and discussion

**Figure 1:**
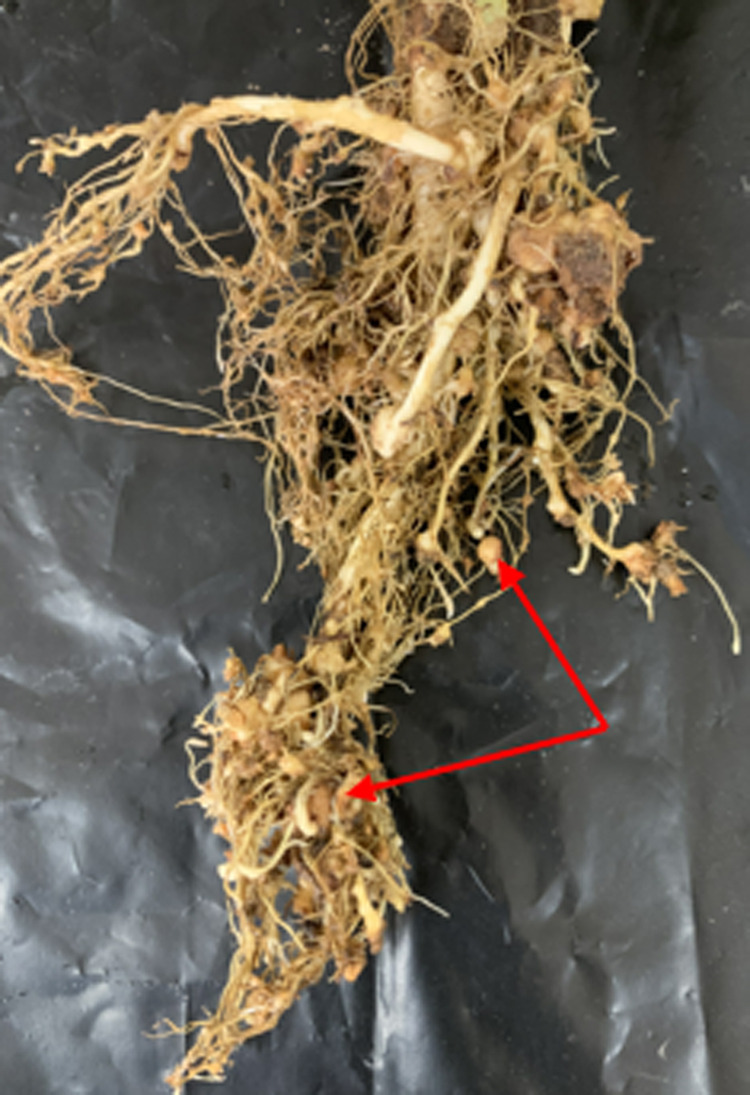
Roots of *Gentiana macrophylla* showing galls induced by *Meloidogyne hapla* (gall and egg mass, arrow).

In the field observation, the plants show symptoms such as slow growth, small and few leaves, and lighter color. Galls and egg masses were visible on roots (Fig. 1), and white, pear-shaped adult females observed inside the roots. The number of galls per plant (*n* = 20) was 53.5 ± 14.5 and egg masses 15.1 ± 3.20. The second-stage juveniles (J2) were collected from the soil in the root zone using standard procedures (Hooper, 1990), population densities of J2 ranged from 225 to 497 per 100 cm^3^.

**Figure 2:**
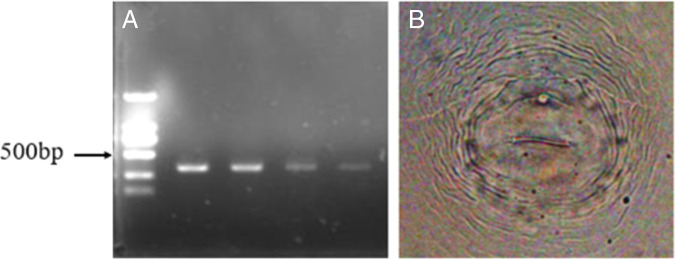
Molecular and morphological identification of *Meloidogyne hapla.* A: PCR amplification products of DNA extracted from *Meloidogyne hapla* using primer set JMV1/JMV hapla. B: Morphological observed on perineal pattern of female. Low range DNA ladder 2,000 bp.

Nematode species were identified using morphological methods and molecular analysis of species-specific PCR. The population showed the following morphometrics: females (*n* = 20) were pear-shaped, 532.62 to 767.45 μm (659.03 ± 72.21) in length, and 243.64 to 557.52 μm (372.79 ± 67.42) in maximum body width, with round perineal pattern, low dorsal arch, and characteristic punctations present near the anus (Fig. 2B). Body length of J2 (*n* = 20) varied from 339.25 to 406.44 μm (372.32 ± 16.92), body width was 13.43 to 18.73 μm (14.48 ± 1.22), stylet length 10.21 to 14.45 μm (13.38 ± 0.87), tail length 36.66 to 51.43 μm (42.43 ± 4.58). These morphological characteristics are consistent with *Meloidogyne hapla* as described by Hunt and Handoo (2009). Species identification was further confirmed by PCR with ITS region primers 18S/26S. PCR produced 768 bp sequences, newly generated sequences (MW897745) compared with available sequences on NCBI. Sequences were 99.35% identical to the MN752202, MT249016, and KJ572385 *M. hapla* sequences. Additionally, a specific band with an expected size of approximately 440 bp was amplified by using the SCAR specific primers (Fig. 2A), the result was matched that of *M. hapla* (Adam et al., 2007). Morphological and molecular characterization supports the identification of the isolate found on *Gentiana macrophylla* as *M. hapla*.

In the experiment to verify Koch postulates, plants were removed from pots and soil gently removed from the roots. A large number of galls and egg masses were found from per plant roots, and isolated the second-stage juveniles (J2) and females. *Gentiana macrophylla* was considered a good host for *M. hapla* in Yulong. To our knowledge, this is the first report of *Gentiana macrophylla* as a host of *M. hapla* in Yulong, China.
